# Short-term side effects of COVID-19 vaccines among healthcare workers: a multicenter study in Iran

**DOI:** 10.1038/s41598-024-54450-w

**Published:** 2024-02-19

**Authors:** Kayvan Mirnia, Elmira Haji Esmaeil Memar, Niyoosha Kamran, Saadollah Yeganedoost, Zeynab Nickhah Klashami, Setareh Mamishi, Shima Mahmoudi

**Affiliations:** 1 Department of Neonatology, Pediatrics Center of Excellence, Children’s Medical Center, Tehran University of Medical Sciences, Tehran, Iran; 2 Pediatrics Center of Excellence, Children’s Medical Center, Tehran University of Medical Sciences, Tehran, Iran; 3 Department of Pediatrics, Pediatrics Center of Excellence, Children’s Medical Center, Tehran University of Medical Sciences, Tehran, Iran; 4 Department of Neonatology, Tabriz University of Medical Science, Tabriz, Iran; 5 Endocrinology and Metabolism Research Institute (EMRI), Tehran University of Medical Sciences, Tehran, Iran; 6 Pediatric Infectious Disease Research Center, Pediatrics Center of Excellence, Children’s Medical Center Hospital, Tehran University of Medical Sciences, Dr. Gharib Street, Keshavarz Boulevard, Tehran, Iran; 7 Biotechnology Centre, Silesian University of Technology, 44-100 Gliwice, Poland

**Keywords:** COVID-19, Healthcare workers, Side effects, Vaccine, Iran, Microbiology, Health care, Health occupations, Medical research

## Abstract

Since the initiation of the COVID-19 vaccination effort, there has been widespread concern regarding vaccine efficacy and potential side effects. This study aimed to explore the short-term side effects of four available COVID-19 vaccines (Sputnik V, Sinopharm, Oxford–AstraZeneca, and Covaxin) among healthcare workers (HCWs) in Iran. The multicenter study involved 1575 HCWs, with the majority received Sputnik V (74.1%), followed by Covaxin (15.6%), Sinopharm (6.4%), and Oxford-AstraZeneca (3.8%). The prevalence of at least one side effect after the first and second dose COVID-19 vaccine was 84.6% and 72.9%, respectively. The common side effects (presented in > 50% of the study participants) after the first dose of the vaccine were injection site pain (61.7%), myalgia (51.8%), and muscle pain (50.9%). The most reported side effects after the second dose of the vaccine were injection site pain (26.8%), myalgia (15.8%), fever (10.3%), headache (9.9%), and chills (9.2%). In conclusion, according to the COVID-19 vaccine type, different side effects might occur following the first and second doses of vaccination. These findings assist in addressing the ongoing problems of vaccination hesitancy which has been driven by widespread worries about the vaccine safety profile.

## Introduction

The novel virus from the Coronaviridae family, named severe acute respiratory syndrome coronavirus 2 (SARS-CoV-2), was first discovered in December 2019 in Wuhan, China^1^. In parallel with the imposed restrictions to prevent viral spread, researchers sped up the development of vaccines to prevent or restrict potential viral damage. COVID-19 vaccine development has utilized diverse platforms, ranging from conventional methods such as inactivated and inactivated with adjuvant vaccines, along with live-attenuated vaccines. Furthermore, innovative strategies have been implemented, including reversed vaccination approaches like recombinant subunit vaccines, as well as advanced techniques employing vector delivery systems, RNA-based vaccines, and DNA-based vaccines^2^.

In Iran, vaccination against COVID‐19 began on February 9, 2021, and was initiated focusing on healthcare workers (HCWs)^3,4^. Sputnik V (Gam-COVID-Vac, Russia, rAd26 and rAd5 viral vectors), Sinopharm (BBIBP-CorV, China, inactivated whole virus), Oxford-AstraZeneca (AZD1222 or ChAdOx1 nCoV-19, South Korea, viral vector), and Covaxin (BBV152, Bharat Biotech, India, inactivated whole virus) were the most frequently administered vaccines in Iran^3,5^.

Following the implementation of the COVID-19 vaccination program, public concern regarding vaccine effectiveness and side effects has increased, affecting the overall acceptance rates of the vaccines^6,7^.

The rapid development of COVID-19 vaccination, the rapid progress in developing COVID-19 vaccines, combined with uncertainties about their safety, is a major factor contributing to the diminished trust in COVID-19 immunization efforts^2,8^. Although the majority of vaccination side effects are mild, a few rare and serious adverse events specific to each type of vaccine have been reported^3,9^. Therefore, post-vaccination surveillance is necessary to provide the necessary measures to deal with short, mid, and long-term complications^10^.

The aim of this study was to investigate the short side effects of four common available COVID-19 vaccines (Sputnik V, Sinopharm, Oxford–AstraZeneca, and Covaxin) in HCWs in four Iranian hospitals.

## Subjects and methods

### Study design and subjects

This multicenter cross-sectional study was carried out from February 1, 2020 to June 31, 2021. This study focused on HCWs of four hospitals, including Children’s Medical Center, a hub of excellence in pediatrics in Tehran, Iran; Imam Khomeini Hospital in Tabriz, Iran; Al-Zahra Maternity Hospital; and Um-Al-Banin Hospital in Mashhad, Iran.

All workers in hospitals, including nurses, administrative personnel, service personnel, physicians, and medical students, who were vaccinated as part of the HCW vaccination program, were eligible for participation in the study.

Prior to participation, informed consent was obtained from all subjects. This study was based on ethical principles and standards for conducting medical research in Iran, and it was ethically approved by Tehran University of Medical Sciences committee **(**IR.TUMS.CHMC.REC.1400.105). The research was conducted in accordance with relevant guidelines and regulations. Inclusion criteria required individuals to be vaccinated and willing to complete the questionnaire. Participants were asked to report any side effects experienced after the first and/or second doses of the vaccine separately. Furthermore, each respondent was allowed to complete the questionnaire only once. Individuals who had received a vaccine other than Sputnik V, Sinopharm, Oxford–AstraZeneca, or Covaxin were excluded from the study.

### Data collection

The study employed a structured questionnaire comprising three sections. The first part is about demographic information such as sex, age, medical history, the type of vaccination status against the COVID-19 vaccine, injection dates, and history of COVID-19 after vaccination. The second part focused on the general side effects of vaccines on several organ systems, including the skeletal, muscular, circulatory, digestive, neurological, and integumentary systems. Participants were requested to specify the onset and duration of each side effect following the first dose, second dose, or both doses of the vaccination.

### Statistical analysis

Statistical analysis was performed using SPSS software version 20.0 (IBM SPSS Statistics for Windows, Armonk, NY: IBM Corp). Descriptive data were shown as numbers and percentages. Chi-square and Fisher’s exact tests were used to find the association among the categorical variables and check the significance level at *P* value < 0.05.

## Results

The study included 1575 healthcare workers (HCWs), with a predominant representation of females (78.5%). Table 1 provides a comprehensive summary of the participants’ general demographic characteristics and medical history. The participants were categorized based on the age group: between 20 and 30 years old (n = 400, 25.4%); 30 and 40 years (n = 562, 35.7%); 40 and 50 years (n = 409, 26%); 50 and 60 (n = 179, 11.4%); and more than > 60 years of age (n = 25, 1.6%). The study shows the age group of 30–40 years in women and 40–50 years in men has the highest percentage.

**Table 1:** Demographic characteristics of all participants (N = 1575).

Parameters	Frequency	Percent
Gender		
Male	338	21.5
Female	1237	78.5
Age		
20–30 year	400	25.4
30–40 year	562	35.7
40–50 year	409	26
50–60 year	179	11.4
> 60 year	25	1.6
Smoking history		
Non-smokers	1494	94.9
Smoker	62	3.9
NA	19	1.2
Health status		
History of underlying disease	305	19.4
History of allergy	244	15.5
History of COVID-19	610	38.7
Treatment (n = 605)		
Admission to an intensive care unit	3	2
Hospitalization	27	1.7
Outpatient treatment	575	36.5
Type of COVID-19 vaccine		
Oxford-AstraZeneca	556	35.3
COVAXIN	134	8.5
Sinopharm	207	13.1
Sputnik V	678	43
Doses frequency		
First dose	969	61.5
Second dose	600	38.1

Totally, 62 (3.9%) of the cases were smokers, 244 participants (15.5%) had allergies, and 19.4% of individuals (n = 305) had comorbidities. Hypertension (46%), diabetes mellitus (5.4%) and asthma (5.4%) were the most prevalent comorbid conditions. Moreover, 39% of participants (n = 610) had a history of COVID-19 during the pandemic.

Regarding the type of vaccine received among the 4 vaccines, Sputnik V was the predominant choice among women, while men predominantly received Oxford-AstraZeneca. The majority of the participants received Sputnik V (43%), Oxford-AstraZeneca (35.3%), followed by Sinopharm (13.2%), and Covaxin (8.5%), respectively.

### Side effects profile

The side effects profile indicated that the majority of side effects occurred within 24 h of vaccine administration (54.6%), with an additional 20.3% occurring between 24 and 48 h after injection. Post-vaccine COVID-19 infection was reported in 12 cases (4.2%). Post-vaccine COVID-19 infection following vaccination with Oxford-AstraZeneca, Sputnik V, Covaxin, and Sinopharm was 5%, 4.5%, 3.6, and 2%, respectively (Table 2).

**Table 2:** Examining the frequency of different types of COVID-19 vaccines received with the time interval of side effects after the first and second doses of the vaccine.

Parameters	Oxford-AstraZeneca	Covaxin	Sinopharm	Sputnik V	*P* value
Post-vaccine COVID-19 infection					
Yes	4 (4.5%)	1 (3.6%)	1 (2.0%)	6 (5.0%)	0.8
No	85 (95.5%)	27 (96.4%)	48 (98.0%)	114 (95.0%)	
	N = 89	N = 28	N = 49	N = 120	
Onset of side-effects after dose 1					
Within 24 h after injection	381 (75.4%)	43 (40.6%)	89 (70.1%)	347 (63.0%)	< 0.001*
Within 24–48 h after injection	99 (19.6%)	46 (43.4%)	22 (17.3%)	153 (27.8%)	
Within 1 week after injection	21 (4.2%)	12 (11.3%)	11 (8.7%)	32 (5.8%)	
Within 2 weeks after injection	4 (0.8%)	4 (3.8%)	3 (2.4%)	11 (2.0%)	
Within 1–2 weeks after injection	0 (0.0%)	1 (0.9%)	2 (1.6%)	8 (1.5%)	
	N = 505	N = 106	N = 127	N = 551	
Onset of side-effects after dose 2					
Within 24 h after injection	3 (60.0%)	37 (46.8%)	13 (76.5%)	244 (71.1%)	0.006*
Within 24–48 h after injection	1 (20.0%)	32 (40.5%)	2 (11.8%)	77 (22.4%)	
Within 1 week after injection	1 (20.0%)	9 (11.4%)	1 (5.9%)	13 (3.8%)	
Within 2 weeks after injection	0 (0.0%)	0 (0.0%)	0 (0.0%)	4 (1.2%)	
Within 1–2 weeks after injection	0 (0.0%)	1 (1.3%)	1 (5.9%)	5 (1.5%)	
	N = 5	N = 79	N = 17	N = 343	

*P value < 0.05 has been considered significant.

Table 3 outlines the most common adverse effects associated with COVID-19 vaccines. The prevalence of side effects after the first and second doses was 75.6% and 71.8%, respectively.

**Table 3:** Side effects experienced after the first and second doses of COVID-19 vaccine.

Side effect	Dose 1						Dose 2					
	AstraZeneca	Covaxin	Sinopharm	Sputnik V	All	*p* value	AstraZeneca	Covaxin	Sinopharm	Sputnik V	All	*p* value
	N = 556	N = 134	N = 207	N = 678	N = 1575		N = 24	N = 97	N = 40	N = 462	N = 623	
Pain at the injection site	442 (79.5%)	78 (58.2%)	88 (42.5%)	364 (53.7%)	972 (61.7)	< 0.001*	3 (12.5%)	56 (57.7%)	10 (25.0%)	195 (42.2%)	264 (42.4)	< 0.001*
Myalgia	379 (68.2%)	60 (44.8%)	54 (26.1%)	323 (47.7%)	816 (51.8)	< 0.001*	4 (16.7%)	44 (45.4%)	5 (12.5%)	196 (42.4%)	249 (40)	< 0.001*
Fever	366 (65.8%)	29 (21.6%)	21 (10.1%)	267 (39.4%)	683 (43.4)	< 0.001*	5 (20.8%)	16 (16.5%)	1 (2.5%)	140 (30.3%)	162 (26)	< 0.001*
Headache	309 (55.6%)	37 (27.6%)	35 (16.9%)	221 (32.6%)	602 (38.2)	< 0.001*	4 (16.7%)	30 (30.9%)	2 (5.0%)	120 (26.0%)	156 (25)	0.009*
Chills	337 (60.6%)	21 (15.7%)	10 (4.8%)	216 (31.9%)	584 (37.1)	< 0.001*	3 (12.5%)	8 (8.2%)	0 (0.0%)	134 (29.0%)	145 (23.3)	< 0.001*
Nausea	121 (21.8%)	12 (9.0%)	8 (3.9%)	63 (9.3%)	204 (13)	< 0.001*	0 (0.0%)	9 (9.3%)	1 (2.5%)	40 (8.7%)	50 (8)	0.2
Redness and inflammation	59 (10.6%)	6 (4.5%)	4 (1.9%)	43 (6.3%)	112 (7.1)	< 0.001*	0 (0.0%)	5 (5.2%)	0 (0.0%)	21 (4.5%)	26 (4.2)	0.3
Cough	56 (10.1%)	4 (3.0%)	10 (4.8%)	17 (2.5%)	87 (5.5)	< 0.001*	0 (0.0%)	6 (6.2%)	0 (0.0%)	21 (4.5%)	27 (4.3)	0.2
Shortness of breath	42 (7.6%)	5 (3.7%)	3 (1.4%)	23 (3.4%)	73 (4.6)	< 0.001*	1 (4.2%)	5 (5.2%)	1 (2.5%)	13 (2.8%)	20 (3.2)	0.6
Vomiting	24 (4.3%)	2 (1.5%)	0 (0.0%)	12 (1.8%)	38 (2.4)	0.002*	0 (0.0%)	3 (3.1%)	0 (0.0%)	8 (1.7%)	11 (1.8)	0.5
Olfactory disorder	10 (1.8%)	2 (1.5%)	1 (0.5%)	8 (1.2%)	21 (1.3)	0.5	0 (0.0%)	1 (1.0%)	0 (0.0%)	2 0.4%)	3 (0.5)	0.8
Runny nose	0 (0.0%)	0 (0.0%)	1 (0.5%)	2 (0.3%)	3 (0.2)	0.4	0 (0.0%)	0 (0.0%)	0 (0.0%)	2 (0.4%)	2 (0.3)	0.8
Other complications	71 (12.8%)	13 (9.7%)	13 (6.3%)	65 (9.6%)	162 (10.3)	0.05*	1 (4.2%)	5 (5.2%)	0 (0.0%)	22 (4.8%)	28 (4.5)	0.5
No side effect	27 (4.9%)	23 (17.2%)	73 (35.3%)	117 (17.3%)	240 (15.2)	< 0.001*	15 (62.5%)	13 (13.4%)	24 (60.0%)	117 (25.3%)	169 (27.1)	< 0.001*

*P value < 0.05 has been considered significant.

### Side effects after the first dose

Overall, the common side effects (presented in > 50% of the study participants) after the first dose of vaccine were injection site pain (61.7%) and myalgia (51.8%), while a moderately common side effect (presented in 30–50% of the study participants) were fever (43.4%), headache (38.2%), and chills (37.1%), respectively. Less common side effects, occurring in a few participants, involved tachycardia, dizziness, diarrhea, restlessness, chest pain, sweating, flushing, abdominal pain, itching, weakness, hypotension, sore throat, and pain in the bones and joints, pelvis, and hands.

### Side effects after the second dose

Of the 1575 participants who received the first dose, 623 (39.5%) received the second dose of COVID-19 vaccines. Of these, the majority of the participants received Sputnik V (74.1%), followed by Covaxin (15.6%), Sinopharm (6.4%), and Oxford-AstraZeneca (3.8%).

The number of cases with no side effects after the second was increased (n = 169, 27.1%). The most reported side effects after the second dose of vaccine were injection site pain (42.4%), myalgia (40%), fever (26%), headache (25%), and chills (23.3%).

Notably, no significant differences were identified in terms of sex, comorbidities, the time interval between contracting COVID-19 and receiving the vaccine, complications from the injection after the first and second doses, and post-vaccine COVID-19 infection (*P* value > 0.05). Furthermore, there was no significant association between the individual’s comorbidities and vaccine complications after the first and second doses of the vaccine (*P* value > 0.05).

## Association of side effects among types of vaccines

### Side effects after the first dose

#### Oxford-AstraZeneca vaccine

The most common adverse effects of Oxford-AstraZeneca vaccine after injection were pain at the injection site (79.5%), myalgia (68.2%), fever (65.8%), chill (60.6%), and headache (55.6%). Only 4.9% of the cases reported no side effects following the first vaccine dose.

#### Sputnik V

First-dose adverse events following immunization with sputnik V was pain at the injection site (58.2%), myalgia (47.7%), fever (39.4%), headache (32.6%), and chill (31.9%); 17.3% reported no side effects.

#### Covaxin

The most common adverse effects of Covaxin vaccine after injection were pain at the injection site (79.5%), myalgia (44.8%), headache (27.6%), and fever (21.6%); 17.3% reported no side effects.

#### Sinopharm

Nearly one-third of the cases reported no side effects (35.3%). The most common adverse effects of the Sinopharm vaccine after injection were pain at the injection site (79.5%), and myalgia (26.1%).

### Side effects after the second dose

#### Oxford-AstraZeneca

Overall, the number of adverse events following the second dose of COVID-19 vaccination were lower than the side effects following the the first dose vaccination.

A majority of cases (62.5%) reported no side effects following the second vaccine dose, with fever and myalgia reported in only 20.8% and 16.7% of cases, respectively.

#### Sputnik V

The most common adverse effects of Sputnik V vaccine after the second injection were myalgia (42.4%), and pain at the injection site (42.2%), with 25% of cases reported no side effects.

#### Covaxin

Second-dose adverse events following immunization with Covaxin were pain at the injection site (57.7%) and myalgia (45.5%), while 13.4% reported no side effects.

#### Sinopharm

Sixty percent of cases vaccinated with Sinopharm showed no adverse effects following the second injection.

Regarding the first dose vaccination, fever, nausea, chills, vomiting, headache, pain at the injection site, shortness of breath, cough, redness and inflammation, and myalgia were significantly associated with the Oxford-AstraZeneca vaccine (Table 3). On the other hand, side effects following the second dose vaccination, including fever, chills, and headache, were significantly associated with the Sputnik V vaccine, while headache, and myalgia were significantly associated with the Covaxin and Sputnik V vaccines, respectively.

## Discussion

To the best of our knowledge, this study presents the most comprehensive analysis of the safety profile of COVID-19 vaccines in HCWs in Iran. All COVID-19 vaccines including Oxford-AstraZeneca, Sputnik V, Sinopharm, and Covaxin exhibited side effects particularly within the initial 24 h after vaccine administration. The prevalence of at least one side effect after the first and second dose of the COVID-19 vaccine was 84.6% and 72.9%, respectively.

Similar to previous studies, females were more likely to experience side effects than males. The higher frequency of side-effects among females can be explained by these biological mechanisms, intense cellular and humoral immune responses to vaccinations^11,12^. However, there is some reports that have reported higher side effects among males compared to females^13–16^.

In our study, similar to previous reports, the majority of reported adverse effects following COVID-19 immunization were mild to moderate and generally resolved within a few days following immunization^3,8,17–21^. The common side effects after the first dose of vaccine were injection site pain (61.7%), myalgia (51.8%), fever (43.4%), headache (38.2%), and chills (37.1%). The most reported side effects after the second dose of vaccine were injection site pain (26.8%), myalgia (15.8%), fever (10.3%), headache (9.9%), and chills (9.2%).

The majority of these side effects manifested within 24 h following vaccination. Consistent with prior studies, fever, chills, headache, myalgia, tiredness, and pain at the injection site were identified as the most common after COVID-19 vaccinations^22,23^. In Xia et al.^24^ study, injection site pain was the most common adverse reaction, followed by mild and self-limiting fever, with no noted severe adverse reactions.

Regarding the first dose vaccination, fever, nausea, chills, vomiting, headache, pain at the injection site, shortness of breath, cough, redness and inflammation, and myalgia were significantly associated with the Oxford-AstraZeneca vaccine. This aligns with previous reports that predominantly highlighted local and systemic side effects, particularly after the first dose of the Oxford-AstraZeneca vaccine^12,25,26^.

Consistent with other findings,, the adverse effects in the second dose were fewer than in the first dose^26^. Side effects following the second dose vaccination, including fever, chills, and headache were significantly associated with the Sputnik V vaccine, while headache and myalgia were significantly associated with the Covaxin and Sputnik V vaccines.

Notably, most of the adverse effects were observed following the first dosage of Oxford-AstraZeneca (95.1%), and there was an increased proportion of cases reporting no side effects after the second dose (62.5%). This elevated occurrence of adverse effects following the first dose of Oxford-AstraZeneca may indicate hesitancy towards receiving the second dose. Vaccine safety concerns^27–29^, including fears of potential side effects and a lack of trust in vaccine creation and distribution processes, have been reported as common reasons for HCWs to hesitate in accepting the COVID-19 vaccine^30^.

Approximately 15.1% and 27.1% of participants reported no side effects after the first dose and second dose of the COVID-19 vaccination, respectively. In the study reported by Jordanian HCWs, 18% and 31% of them reported no side effects following the first and second dose of the COVID-19 vaccination^25^.

In our study, a majority of HCWs (79.5%) had pain at the injection site following the first dose of the AstraZeneca vaccination. A study conducted in Ethiopia found that 75.8% of HCWs who received the AstraZeneca vaccine reported injection site symptoms^21^. Additionally, 55.6% of HCWs in our study reported developing a headache after receiving the AstraZeneca COVID-19 vaccine, consistent with findings in other studies^21,31^. The frequency of myalgia following the first-dose administration of AstraZeneca was 68.2%, which was higher than previous reports^21,25^.

In this study, the incidence of post-vaccine COVID-19 infection was higher than what has been reported in previous studies^16^. The effectiveness of the Oxford-AstraZeneca vaccine in preventing COVID-19 was reported to be 70.4%, and for Sinopharm, it ranged between 50 and 78%^25,31^. Several limitations should be acknowledged in our study. Firstly, the side effect profile was based on self-reported data, which introduces the potential for recall bias and subjective interpretation. Participants may not accurately recall or may perceive side effects differently, impacting the reliability of the reported information. Secondly, the study did not evaluate the severity of side effects, limiting our understanding of the intensity and impact of adverse reactions. Assessing the severity could provide a more comprehensive picture of the overall safety profile of the vaccines. Thirdly, the long-term effects of the observed side effects were not investigated in this study. Understanding the duration and persistence of adverse reactions is crucial for a comprehensive assessment of vaccine safety. Lastly, the absence of specific information regarding the identification, measurement, or control of potential covariates in the statistical analysis represents a limitation. This study did not explore the potential influence of pre-existing medical conditions or medications on the occurrence and severity of side effects.

## Conclusion

The type of COVID-19 vaccine administered appears to influence the occurrence and nature of side effects following the first and second doses of vaccination. Our study indicates that the side effects associated with COVID-19 vaccines among HCWs were predominantly mild in severity. Notably, after the initial vaccination, Oxford-AstraZeneca, Sputnik V, and Covaxin resulted in a higher incidence of side effects, while the second dose of Sputnik V and Covaxin elicited more side effects. It is noteworthy that although a substantial frequency of adverse effects was noted following the first dose of Oxford-AstraZeneca (95.1%), a considerable proportion of individuals reported no side effects after receiving the second dose of the vaccine (62.5%). These findings contribute valuable insights to address the persistent challenges of vaccination hesitancy, which stem from widespread concerns about the safety profile of COVID-19 vaccines.

## Data Availability

The datasets used and/or analyzed during the current study are available from the corresponding author upon reasonable request.

## References

[1] Huang C (2020). Clinical features of patients infected with 2019 novel coronavirus in Wuhan, China. Lancet.

[2] Wibawa T (2021). COVID-19 vaccine research and development: Ethical issues. Trop. Med. Int. Health.

[3] Pourakbari B (2022). Evaluation of response to different COVID-19 vaccines in vaccinated healthcare workers in a single center in Iran. J. Med. Virol..

[4] Abdolsalehi MR (2022). SARS-CoV-2 transmission among healthcare workers in Iran: An urgent need for early identification and management. Infect. Disord. Drug Targets.

[5] Oghazian S, Tavanaei Tamanaei T, Haghighi R, Faregh M, Oghazian MB (2023). Side effects of Sputnik V, Oxford-AstraZeneca, Sinopharm, and Covaxin and their associations with other variables among healthcare workers of a tertiary hospital in Iran. Int. Immunopharmacol..

[6] Joshi A (2021). Predictors of COVID-19 vaccine acceptance, intention, and hesitancy: A scoping review. Front. Public Health.

[7] Mirahmadizadeh A (2022). COVID-19 vaccine acceptance and its risk factors in iranian health workers 2021. Iran J. Med. Sci..

[8] Pordanjani SR (2022). A comprehensive review on various aspects of SARS-CoV-2 (COVID-19) vaccines. Int. J. Prev. Med..

[9] Nguyen SV (2023). Side effects following first dose of COVID-19 vaccination in Ho Chi Minh City, Vietnam. Hum. Vaccines Immunother..

[10] Khezri R, Golfiroozi S, Nikbakht H-A, Maleki Z, Ghelichi-Ghojogh M (2022). COVID-19 vaccine and the necessity to identify its side effects. J. Health Sci. Surveill. Syst..

[11] Nachtigall I (2022). Effect of gender, age and vaccine on reactogenicity and incapacity to work after COVID-19 vaccination: A survey among health care workers. BMC Infect. Dis..

[12] Zare Z (2022). Signs, symptoms, and side-effects presented by different types of COVID-19 vaccines: A prospective cohort study. Life (Basel).

[13] Elnaem MH (2021). COVID-19 vaccination attitudes, perceptions, and side effect experiences in Malaysia: Do age, gender, and vaccine type matter?. Vaccines.

[14] Mohamed MS (2023). A first report on side-effects of COVID-19 vaccines among general population in Sudan: A cross-sectional analysis. Vaccines (Basel).

[15] Hatmal MM (2022). Reported adverse effects and attitudes among arab populations following COVID-19 vaccination: A large-scale multinational study implementing machine learning tools in predicting post-vaccination adverse effects based on predisposing factors. Vaccines (Basel).

[16] Al Bahrani S (2021). Safety and reactogenicity of the ChAdOx1 (AZD1222) COVID-19 vaccine in Saudi Arabia. Int. J. Infect. Dis..

[17] Xia S (2021). Safety and immunogenicity of an inactivated SARS-CoV-2 vaccine, BBIBP-CorV: A randomised, double-blind, placebo-controlled, phase 1/2 trial. Lancet Infect. Dis..

[18] Ella R (2021). Safety and immunogenicity of an inactivated SARS-CoV-2 vaccine, BBV152: A double-blind, randomised, phase 1 trial. Lancet Infect. Dis..

[19] Ramasamy MN (2021). Safety and immunogenicity of ChAdOx1 nCoV-19 vaccine administered in a prime-boost regimen in young and old adults (COV002): A single-blind, randomised, controlled, phase 2/3 trial. Lancet.

[20] Logunov DY (2021). Safety and efficacy of an rAd26 and rAd5 vector-based heterologous prime-boost COVID-19 vaccine: An interim analysis of a randomised controlled phase 3 trial in Russia. Lancet.

[21] Solomon Y, Eshete T, Mekasha B, Assefa W (2021). COVID-19 vaccine: Side effects after the first dose of the Oxford Astrazeneca vaccine among health professionals in low-income country: Ethiopia. J. Multidiscip. Healthc..

[22] McDonald I, Murray SM, Reynolds CJ, Altmann DM, Boyton RJ (2021). Comparative systematic review and meta-analysis of reactogenicity, immunogenicity and efficacy of vaccines against SARS-CoV-2. NPJ Vaccines.

[23] Anderson EJ (2020). Safety and immunogenicity of SARS-CoV-2 mRNA-1273 vaccine in older adults. N. Engl. J. Med..

[24] Xia S (2020). Effect of an inactivated vaccine against SARS-CoV-2 on safety and immunogenicity outcomes: Interim analysis of 2 randomized clinical trials. JAMA.

[25] Abu-Hammad O (2021). Side effects reported by Jordanian healthcare workers who received COVID-19 vaccines. Vaccines (Basel).

[26] Enayatrad M (2023). Reactogenicity within the first week after Sinopharm, Sputnik V, AZD1222, and COVIran barekat vaccines: Findings from the Iranian active vaccine surveillance system. BMC Infect. Dis..

[27] Sharma A, Jain M, Vigarniya M (2022). Acceptance and adverse effects following COVID-19 vaccination among the health care workers at a health care centre in the most backward district of India. J. Fam. Med. Prim. Care.

[28] Shaw J (2021). Assessment of US healthcare personnel attitudes towards coronavirus disease 2019 (COVID-19) vaccination in a large university healthcare system. Clin. Infect. Dis..

[29] Shekhar R (2021). COVID-19 vaccine acceptance among health care workers in the United States. Vaccines (Basel).

[30] Elharake JA (2021). COVID-19 vaccine acceptance among health care workers in the Kingdom of Saudi Arabia. Int. J. Infect. Dis..

[31] Voysey M (2021). Safety and efficacy of the ChAdOx1 nCoV-19 vaccine (AZD1222) against SARS-CoV-2: An interim analysis of four randomised controlled trials in Brazil, South Africa, and the UK. Lancet.

